# Physiological and Immune Functions of Punicalagin

**DOI:** 10.3390/nu13072150

**Published:** 2021-06-23

**Authors:** Eva Venusova, Adriana Kolesarova, Pavel Horky, Petr Slama

**Affiliations:** 1 Department of Animal Morphology, Physiology and Genetics, Faculty of AgriSciences, Mendel University in Brno, Zemedelska 1, 613 00 Brno, Czech Republic; eva.venusova@mendelu.cz; 2 Department of Animal Physiology, Faculty of Biotechnology and Food Sciences, Slovak University of Agriculture in Nitra, Tr. A. Hlinku 2, 949 76 Nitra, Slovakia; adriana.kolesarova@uniag.sk; 3 Department of Animal Nutrition and Forage Production, Faculty of AgriSciences, Mendel University in Brno, Zemedelska 1, 613 00 Brno, Czech Republic; pavel.horky@mendelu.cz

**Keywords:** punicalagin, ellagic acid, apoptosis, proliferation, immune cells, metabolism

## Abstract

The aim of this publication is to compile a summary of the findings regarding punicalagin in various tissues described thus far in the literature, with an emphasis on the effect of this substance on immune reactions. Punicalagin (PUN) is an ellagitannin found in the peel of pomegranate (*Punica granatum*). It is a polyphenol with proven antioxidant, hepatoprotective, anti-atherosclerotic and chemopreventive activities, antiproliferative activity against tumor cells; it inhibits inflammatory pathways and the action of toxic substances, and is highly tolerated. This work describes the source, metabolism, functions and effects of punicalagin, its derivatives and metabolites. Furthermore, its anti-inflammatory and antioxidant effects are described.

## 1. Introduction

Eating a diet involving increased amounts of fruits and nuts is very beneficial for human health and can serve for the prevention of various diseases. Currently, there is a growing interest in research regarding the components of pomegranate (*Punica granatum*) in terms of healthy nutrition and medicine. It is used mainly in Asian countries in traditional medicine and has long been considered as a blood tonic [1]. However, it is now also widely grown in parts of Southwest America, Arizona, Mexico, California and Africa [2]. 

The inclusion of drinking pomegranate juice is associated with health benefits, which can partly reduce inflammatory processes, inhibit and prevent carcinogenesis, alleviate diabetes and promote wound healing via antioxidant activity [3,4,5]. These benefits are attributed to polyphenols, which consist mainly of hydrolyzed tannins [6]. The most abundant ingredient in pomegranate peel is punicalagin (PUN). Gil et al. [7] state that 87% of the antioxidant activity measured in pomegranate juice is due to its hydrolysable tannin content, including PUN. It can reach concentrations of >2 g/L in juice [8]. It consists of glucose, which is located in the center of PUN. Glucose exists in α- and β-anomeric forms esterified with ellagic acid (EA), gallic acid dimers, gallagic acid and EA dimers [6,9].

Tannins are generally known for their ability to bind to molecules, such as proteins, polysaccharides, metals and DNA [10]. Kulkarni et al. showed that PUN has a high affinity for metal ions and bovine serum albumin but very weak and non-specific binding to DNA [11].

Several in vitro studies have confirmed a wide range of biological activities for this substance. PUN has been shown to stimulate apoptosis in promyelocytic leukemia cells, colon cancer cell lines and glioma cells, in addition to inhibiting cancer cell proliferation and modulating inflammatory subcellular signaling pathways [12,13]. PUN can also be very useful as a broad-spectrum antiviral agent to reduce recurrent disease-causing viruses (HCV, RSV and HSV-1), which are known to use viral glycoprotein interactions with cell surface glycosaminoglycans to enter the host cell [14]. 

In addition, PUN, EA and its derivatives have been shown to have antimutagenic, antioxidant properties and can protect DNA [15]. In addition, it is used to decrease the symptoms of cardiovascular disease, diabetes, diarrhea, bronchitis, asthma, bleeding disorders, fever, cough, inflammation, atherosclerosis, acquired immunodeficiency syndrome, mouth lesions, ulcers, skin lesions, malaria, prostate cancer, hypertension, periodontal disease, hyperlipidemia, male infertility, vaginitis, erectile dysfunction, obesity, pediatric cerebral ischemia and Alzheimer’s disease [2]. 

## 2. Methods

The keywords “punicalagin”, “ellagitannins”, “effect of punicalagin”, “metabolism of ellagitannins”, “immune function of punicalagin”, “pomegranate”, “bioavailability of punicalagin” and “pharmacological effect of punicalagin” were used to search publications that had been entered into online databases, such as the Web of Science, Pubmed and Science Direct. A total of 209 articles were found by searching for keywords in the Web of Science online database. Obtained articles were subsequently manually searched, and non-matching articles were discarded.

## 3. Metabolism and Bioavailability of Punicalagin

The metabolism of ellagitannins in the gastrointestinal tract is complicated. All ellagitannins, including PUN, have the same ability to be hydrolyzed in the small intestine to EA [16]. However, the bioavailability of ellagitannins and EA is very low, and compounds that are unable to be absorbed are then further metabolized [17]. EA is metabolized to catabolic intermediates by a series of decarboxylation reactions performed by intestinal microbiota. Bacteria produce dibenzopyran-6-one derivatives or urolithins [18]. 

Due to the complex catabolism of these substances, the real bioactive molecules could be urolithins rather than PUN or EA. Urolithins are formed from EA by the loss of one of the two lactones and the gradual removal of hydroxyl groups. From a chemical point of view, they can be said to be a combination of coumarin and isocoumarin (benzocoumarins) [17]. 

The end products are urolithin A, urolithin B and isourolithin A. Researchers found that not every individual was able to produce the final metabolites of urolithin due to differences in intestinal microbiota. Three different metabotypes of urolithin (UM) have been identified, namely UM-A (individuals who are only able to produce urolithin A), UM-B (individuals who are able to produce urolithin A, urolithin B and also isourolithin A) and UM-0 [19,20]. 

This classification is defined on the basis of the type of urolithin present in the urine 24 h after ingestion [18]. The components of the intestinal microbiota involved in the conversion of EA to intermediate urolithins in humans are bacteria of the genus Gordonibacter [21,22]. A positive correlation of these fecal bacteria with urolithin A content in feces and urine was found [23]. The amount of Gordonibacter bacteria was found to be higher in individuals with urolithin metabotype A compared with individuals with urolithin metabotype B or 0 [24]. Genes encoding enzymes involved in the metabolism of EA to urolithins have also been investigated. Candidate enzymes that could be involved in the EA metabolism are tannin acetylhydrolase, dehydroxylase and gallate decarboxylase [25].

Due to the rapid conversion of ellagitannins to EA in the gastrointestinal tract, the relevance of the direct application of ellagitannins, such as PUN, in in vitro cultures may be questioned [18]. The first bioavailability testing of pomegranate ellagitannins was performed on rats and showed that, after the ingestion of large amounts of ellagitannins, metabolites such as urolithins A, B and C, and minor amounts of dimethyl ether-glucuronide of ellagic acid were detected in the plasma and urine [26]. After long-term intake, small amounts of PUN were detected in rats; however, this was not confirmed in further human studies [27]. 

Gonzales et al. conducted research with healthy volunteers who consumed two pomegranate extracts that differed in free EA content. Simultaneously, they simulated gastric and intestinal conditions (pH and protein content) to elucidate various factors involved in the bioavailability of free EA, the role of PUN as a precursor to EA, the EA catabolism to urolithins and the potential impact of pH and protein on PUN and EA solubility and bioavailability [20]. 

Research demonstrated that released EA is detected in blood plasma at a maximum concentration of 100 nM 1 h after administration, even with a high intake of free EA, and remains there for 5–24 h [17,20]. Control tests were performed to identify the impact of the pH and protein content on EA and PUN. The solubility of free EA was found to be pH dependent (pH 7–8) and no significant protein binding was found. In the case of PUN, the solubility was not pH-dependent; however, in the presence of a protein-rich medium, PUN binding to proteins occurred, and it was confirmed that, under these conditions, there was no hydrolysis of PUN and subsequent release of free EA [20].

Although promising therapeutic effects of PUN and EA have been shown in preclinical studies, the solubility of these substances is insufficient to achieve effective use after oral administration. According to the Biopharmaceutical Classification System (BSC), which classifies drugs based on their water solubility and intestinal permeability, EA is classified as a Class IV drug (i.e., a drug with low solubility and permeability). This greatly limits its clinical use [28]. 

An improvement in the bioavailability of PUN and EA can be achieved by reducing the particle size using a micronization technique [29]. Nanoparticles are commonly produced by controlled precipitation, crystallization, high pressure homogenization, wet bead milling and the use of supercritical fluids [30]. Another possible alternative to improve the bioavailability is to encapsulate PUN or EA in biodegradable nanoparticles. The latest, innovative and highly biocompatible EA formulations consist of pectin-dried dispersions, cyclodextrin-based nanosponges, zein nanocapsules, chitosan/alginate microspheres, lactoferrin/chondroitin sulfate nanoparticles and supersaturated self-microemulsification delivery systems [28].

## 4. Methods of Determination PUN and EA

Analytical methods, including gas chromatography, liquid chromatography (HPLC) and mass spectrometry, are widely used to verify PUN and related substances in pomegranate [6]. HPLC is used to separate the components of a sample in order to determine their presence and concentration in the sample or to isolate the individual components of the mixture. Qu et al. developed a method for the determination of ellagic, gallic acid and PUN-A and -B using HPLC. The advantages of the HPLC method are good linearity imaging, double reproducibility, high recovery rate with low limit of detection and quantification. Compared to existing methods, this method offers a significant improvement in sample permeability and allows for the quantification of the four major polyphenolics in a single run [31]. Other analytical methods used included Fourier transform infrared spectroscopy and nuclear magnetic resonance (NMR) spectroscopy [6].

## 5. Anti-Inflammatory and Immunosuppressive Effect 

The inflammatory response is a complex set of interactions between harmful factors and cells that can arise in any tissue due to infectious, toxic or autoimmune damage [32]. An appropriate inflammatory reaction is the body’s defense mechanism, which removes harmful stimuli and initiates the healing process [33]. The incidence of inflammatory diseases has increased worldwide, and they are treated with conventional anti-inflammatory drugs, such as steroids and NSAIDs. 

However, their long-term use can cause a number of side effects, which can be very serious [34]. In the last decade, researchers have studied the components of pomegranates, and they confirmed that their antioxidant, anti-inflammatory and immunosuppressive effects may contribute to treatment due to their pharmacological effects [33,35].

### 5.1. NF-kB and MAPK Activation Inhibitor

Recent studies indicated that PUN inhibits the production of tumor necrosis factor alpha (TNF)-α and interleukin (IL)-1β or IL-6 by macrophages RAW264.7 and in primary human chondrocytes. RAW264.7 macrophages were stimulated with lipopolysaccharide (LPS) [36]. LPS is the most abundant component in the cell wall of Gram-negative bacteria and is a potent activator of macrophages in various cell types that produce a number of proinflammatory mediators, such as NO, PGE2 and interleukins, leading to an acute inflammatory response to pathogens [34,37]. 

Bacterial LPS are widely used in the study of inflammation because they stimulate many inflammatory cytokines, such as TNF-α, IL-1β and IL-6 [37]. The study by Xu et al. [33] and Cao et al. [36] independently found that PUN at the concentration used strongly inhibited LPS-induced NO, PGE2, IL1β, IL-6 and TNF-α secretion in RAW264.7 cells. PUN was also found to attenuate LPS-induced phosphorylation of NF-kB, p38, JNK and ERK MAPK, suggesting that PUN suppresses NF-kB and MAPK signaling pathways to suppress NO, TNF-α and IL-6 induced by LPS. As inflammation is a main etiological factor that is important to many chronic diseases; therefore, this anti-inflammatory activity of PUN has great potential for preventing many diseases and towards health promotion and anti-inflammatory drug development [33,36].

### 5.2. Nuclear Factor of Activated T-Cells Activation Inhibitor

The transcription factor nuclear factor of activated T-cells (NFAT) plays a crucial role in the expression of autocrine growth factor IL-2, which promotes T cell proliferation by interacting with the IL-2 receptor (IL-2R) [38]. As T cell activation is a major point in the development of autoimmune diseases, Lee et al. conducted research into natural products that suppress T cell activity. PUN was found to strongly suppress the immune system due to its inhibitory effect on NFAT activation. PUN reduced IL-2 mRNA and protein expression from anti-CD3/anti-CD28 stimulated mouse spleen CD4+ T cells and suppressed the mixed lymphocyte response (MLR) without exhibiting cytotoxicity to the cells. In vivo treatment with PUN inhibited chronic ear swelling in mice induced by phorbol 12-myristate 13-acetate (PMA) and reduced the infiltration of inflamed tissue by CD3+ T cells [35]. 

### 5.3. Apoptosis, Proliferation and Angiogenesis

Apoptosis is a process of programmed cell death. It commonly occurs during development, aging and as a homeostatic mechanism to maintain cell populations in tissues. It also occurs as a defense mechanism, for example in immune reactions or when cells are damaged by disease or harmful substances [39,40]. PUN is known to stimulate apoptosis in colon cancer cell lines, promyelocytic leukemia cells and glioma cells [41]. The effect of PUN was found in cervical cancer, where PUN stimulated cell apoptosis and blocked cell proliferation by suppressing NF-kB [41,42]. 

The NF-kB signaling pathway is activated in many types of cancer and serves as an inducible modulator of tumorigenesis [43], which is responsible for the transcription and control of genes regulating various cellular functions [41]. NF-kB signaling controls a large number of cellular processes, including immune responses, proliferation, immune cell viability, lymphogenesis and B cell maturation [44]. Huang et al. were the first to report that PUN treatment in osteosarcoma cells significantly inhibited cell proliferation, invasion and apoptosis. Disorders of angiogenesis were also observed after PUN injection [44]. 

Previous research has shown that PUN has the effect of increasing the expression level of the pro-apoptotic marker (Bax) X-protein and down-regulating the protein expression status of antiapoptotic markers (Bcl-XL and Bcl-2) in the prostate cancer cell line LAPC4 [41,45]. Similarly, PUN and its metabolites induce an intrinsic pathway of apoptosis in human colon cancer cells through the down-regulation of Bcl-XL with mitochondrial release of cytochrome c into the cytosol and activation of caspase-9 and caspase-3 [12,39,41].

In addition to regulating cancer cell proliferation and apoptosis, PUN has the effect of reducing the expression of cell cycle proteins, including cyclin A, cyclin B, cyclin D1, cyclin D2 and cyclin E [46,47]. Tang et al. found that the Wnt/β-catenin pathway is associated with cervical cancer. In this study, PUN was found to reduce the expression of β-catenin and its subsequent proteins, including cyclin D1, which is essential for retinoblastoma phosphorylation and its release from the transcription factor E2, leading to cell cycle progression and cell proliferation [12]. 

Treatment of PUN cells leads to many molecular features of apoptosis. Although PUN is able to induce apoptosis, an alternative pathway of cell death—so-called autophagy—is also activated [47]. 

### 5.4. Autophagy

Autophagy is type II cell death. It is a process that ensures a balance between synthesis and degradation, involving the degradation of long-lived intracellular proteins or damaged organelles by the lysosomal apparatus [47]. A study by Wang et al. demonstrated that PUN activates AMP-activated protein kinase (AMPK) and at the same time increases the phosphorylation of cyclin-dependent kinase p27 (Kip1) on Thr 198, which, in turn, leads to the induction of cellular autophagy in human glioma cells via the LKB1-AMPK-p27 signaling pathway [47]. This hypothesis was verified in a study by Liang et al., whose research showed that activation of the LKB1-AMPK pathway increased p27 phosphorylation at Thr 198, leading to p27 stabilization and the subsequent induction of cellular autophagy [48]. 

## 6. Pharmacological Effect of Punicalagin and Metabolites

### 6.1. Antibacterial Effect

Studies have shown antibacterial effects of PUN against Gram-positive and Gram-negative bacteria. PUN and EA have been proven to have antimicrobial activity against, *Staphylococcus aureus*, *Pseudomonas aeruginosa*, *Escherichia coli* and some species of *Clostridia* [49]. PUN has been confirmed to inhibit the growth of cariogenic bacteria at high concentrations; however, at subbactericidal concentrations, it inhibits biofilm development and the production of acidic and extracellular polysaccharides by *Streptococcus mutans*, suggesting that PUN has the potential to prevent tooth decay [50].

### 6.2. Antiviral Effect

Lin et al. performed an extensive analysis of the effect of hydrolysable tannins on a panel of viruses. The route of virus binding, infection entry and spread during treatment with these substances was studied. Antiviral activity was found against viruses known to use cell surface glycosaminoglycans (GAGs) to enter the host cell. The study reported that PUN was effective in suppressing human cytomegalovirus (HCMV) virus, herpes simplex virus (HSV-1), hepatitis C virus (HCV), respiratory syncytial virus (RSV), measles (MV) and dengue virus (DENV), at various concentrations without significant cytotoxicity [14]. Tito et al. even reported in their study that PUN and EA inhibited the interaction between Spike protein and ACE2 and reduced viral 3CL protease activity in vitro, indicating the potential use of pomegranate extract as a prevention and treatment for SARS-CoV-2 disease [51]. 

### 6.3. Antioxidant Activity—Oxidative Stress

The cause of oxidative stress is an imbalance between the accumulation and production of oxygen-reactive species (ROS) in cells and tissues and the ability of the biological system to detoxify these reactive products [52]. As a result of this imbalance, important cellular macromolecules (lipids, carbohydrates, proteins and DNA) are damaged. Oxidative stress is also associated with the manifestation of many chronic diseases, such as metabolic, neurodegenerative, cardiovascular, lung, kidney and cancer diseases [53,54]. Several antioxidants have been described that have a beneficial effect against oxidative stress, including PUN, and metabolized EA and urolithins.

Sun et al. described their research, in which they used different antioxidant assays (ferric reducing antioxidant power and lipid peroxidation) or free radical scavenging assays (DPPH and O_2_^−^). Tests confirmed strong antioxidant capacities of PUN, PL (punicallin) and EA, with differences that could be related to different polymerization and number of unsaturated double bonds [55]. 

Some publications suggested that PUN and PL could have a greater ability to scavenge free radicals than EA due to their high degree of hydroxylation [56]. Results by Sun et al. showed that PL represented the lowest LPO inhibitory capacity of all three compounds. All three compounds showed a strong antioxidant effect; however, their abilities differed in the type of free radicals. EA was more effective than PUN or PL in protecting against oxidative damage in vivo, especially intestinal damage [55]. 

EA-derived urolithins have also been recognized as modulators of oxidative stress. Bialonska et al. performed a test measuring the ability of test compounds (urolithin A; B; C; D; 8-O-methylurolithin A; 8,9-di-O-methylurolithin C; and 8,9-di-O-methylurolithin D) to inhibit intracellular ROS production. The results showed a significant antioxidant effect of urolithins correlated with the number of hydroxyl groups and the lipophilicity of the molecules. The highest antioxidant activity was found for urolithin C (IC_50_ = 0.16 μM) and urolithin D (IC_50_ = 0.33 μM). Urolithin A showed less significant antioxidant activity (IC_50_ = 13.6 μM), while urolithin B and all methylated urolithins did not show any antioxidant activity at all. The results of this study showed that urolithins may have systemic antioxidant effects [57]. 

### 6.4. Hepatoprotective Activity

Fouad et al. found that PUN was able to protect against cyclophosphamide-induced hepatotoxicity (CYP) in rats. CYP is an alkylating nitrogen yperite used as an anticancer and immunosuppressive agent. However, this substance has many toxic effects that inhibit its effectiveness [58]. The metabolites formed from cyclophosphamide in the liver (phosphoramide and acrolein) increase the formation of reactive oxygen species (ROS). 

This leads to oxidative stress and the subsequent activation of the NF-κB signaling pathway regulating the inflammatory cascade and the production of proinflammatory cytokines, i.e., TNF-α and IL-1β [59]. CYP has been shown to cause significant damage to liver tissue and apoptosis and necrosis in liver tissue, respectively. PUN can maintain liver tissue integrity and reduce the liver damage scores to controllable levels [58]. 

In another study, rats were administered acrylamide (ACR), which is commonly used in industries. ACR causes oxidative stress and triggers apoptosis in the brain and liver tissues [60]. After the application of ACR (50 mg/kg/11 days), severe motor disorders were observed in rats, while pretreatment of rats with various doses of PUN, especially 20 mg/kg, reported protective effects against ACR toxicity in the tissues tested. The antioxidant and antiapoptotic attribute of PUN can be regarded as the main mechanisms of protection against ACR-induced toxicity [60]. 

### 6.5. Anti-Diabetic and Anti-Obesity Activity 

Currently, obesity and diabetes have become an increasingly serious health problem for people. A recent study on metabolic diseases, such as diabetes and obesity, showed that PUN, EA and urolithin A had the capability to inhibit enzymes associated with carbohydrate and triglyceride metabolism, such as DPP-4, α-GLU and lipase. During the differentiation of the 3T3-L1 cell line with these polyphenols, the efficiency of these compounds to inhibit adipogenesis as well as the ability to reduce triglyceride accumulation was demonstrated. They have also been shown to have the potential to modulate the expression of genes that regulate fatty acid and glucose metabolism, such as the GLUT4, FABP4, adiponectin and PPARy genes, which are commonly used as markers of adipocyte differentiation [61]. 

The research of Wu et al. demonstrated that PUN and EA remarkably inhibited lipid accumulation in 3T3-L1 adipocytes in a dose-dependent manner. The 3T3-L1 cell line derived from disaggregated Swiss mouse 3T3 embryos is the most widely used and reliable model of adipocyte culture. In this study, they found that 5.24 μg/mL (5 μM) PUN and 4.5 μg/mL (15 μM) EA showed the same inhibitory activities with C75 and EGCG but at much lower concentrations. Thus, PUN and EA showed stronger inhibitory effects than the classical fatty acid synthase (FAS) inhibitors C75, cerulenin and EGCG. As FAS plays a major role in the fatty acid biosynthetic pathway, these findings suggest that PUN and EA are potentially useful in the prevention and treatment of obesity [62].

In a study by Requero et al., six plant extracts were selected for research. The aim was to investigate the metabolic processes involved in the activation of thermogenesis and the increase in mitochondrial respiratory capacity as complementary approaches to increase energy expenditure in obesity-related metabolic changes. The results showed that PUN increased the expression of UCP1, UCP2, BMP8B and CKMT2 in mature adipocytes in line with increased mitochondrial H^+^ leakage and decreased neutral lipid accumulation, suggesting a positive effect on the induction of thermogenesis in white adipose tissue [63].

### 6.6. Anti-Atherosclerotic Activity

Atherosclerosis is a chronic disease due to many factors (e.g., fatty food intake and genetics) that damage the artery wall and vascular function. The disease is characterized by impaired lipid metabolism and endothelial function. It is also one of the major causes of death worldwide. Endothelial dysfunction is characterized by the increased expression of adhesion molecules, such as vascular cell adhesion molecule-1 (VCAM-1) and intercellular adhesion molecule-1 (ICAM-1), allowing the accumulation of monocytes in the subendothelial matrix. Infiltrating monocytes differentiate into macrophages and/or dendritic cells. The intake of native LDL and oxidized low density lipoprotein (oxLDL) by these cells leads to the formation of foam cells, which, in turn, leads to atherosclerosis [64,65]. 

As cholesterol degradation has been found to be indirectly associated with the occurrence of cardiovascular events, research has been conducted that shows a link between cholesterol degradation capacity and cardiovascular mortality. Ellagitannins and their bioactive compounds, which are converted to low molecular weight compounds, including urolithin B, by intestinal microbiota, have been shown to significantly increase the degradation of cholesterol from macrophages. The data from the study demonstrated that urolithin B was able to reduce lipid plaque deposition and modulate the expression of Scavenger Class B receptors type I (SR-BI) and ABCA1 involved in reverse cholesterol transport [66]. 

Cui et al., in their study, investigated whether urolithin A had therapeutic potential in ameliorating atherosclerotic lesions in Wistar rat models. These rats were fed a high cholesterol diet supplemented with vitamin D3 for 12 weeks. Subsequently, the rats were administered urolithin A (3 mg/kg/day) three days before aortic injury. After twelve weeks of urolithin A treatment, there was a significant reduction in the plasma lipid and Ang II levels and an improvement in the aortic lesion compared to the placebo group. There was increased expression of SR-BI, inhibition of p-ERK1/2 and activation of the Nrf-2 signaling pathway. The SR-BI expression was indirectly correlated with Ang II levels [67]. 

In the study by Mele et al., the effects of the metabolites of ellagitannins Uro A, Uro B, Uro C and Uro D as well as their precursor EA, which were investigated at low concentrations of μM (1–10 μM) in two main events leading to the formation of atherosclerotic plaques, namely endothelial activation and the resulting recruitment of circulating monocytes and cholesterol transport and foam cell formation. Of the metabolites tested, Uro C was the most potent with biological activity similar to EA, while Uro A and Uro B were active in combination at 10 μM [64].

## 7. Toxicological Findings and Genotoxicity

There have been, thus far, only a few scientific studies describing the toxicity of the ellagitannins contained in pomegranate. Research in cattle indicates that the intake of large amounts of ellagitannins, especially PUN, was associated with hepatotoxicity and nephrotoxicity [17,68]. In a study of a complex mixture of pomegranate, tannins were recognized as antioxidants but were found to have some genotoxic activity [69]. Labieniec et al. argued that tannic acids, including tannin, gallic and ellagic acids, could contribute to the formation of single-stranded DNA breaks [70]. 

Research conducted on cultured Chinese hamster B14 cells found that the tannin components, which are called antioxidants, can act as prooxidants [70]. The study by Xu et al. stated that PUN had no cytotoxic effect at concentrations from 0 to 400 μM in the treatment of RAW264.7 cells, suggesting that PUN had an inhibitory effect not due to a decrease in cell viability [33]. The repeated oral administration of high doses of pomegranate PUN to rats for 37 days was non-toxic, and it is expected that PUN could be used in humans without bringing about severe toxicity [68]. 

Likewise, the research of Zahin et al. confirmed that, in a genotoxicity study, PUN and EA did not show any mutagenic effect on *Salmonella typhymurium*, on the contrary, they showed protection against DNA damage and high antiproliferative activity. PUN and EA have been shown to have almost similar levels of antimutagenic properties against a number of mutagens and may be promising candidates for future anticancer drugs [15]. 

## 8. Conclusions

**Figure 1:**
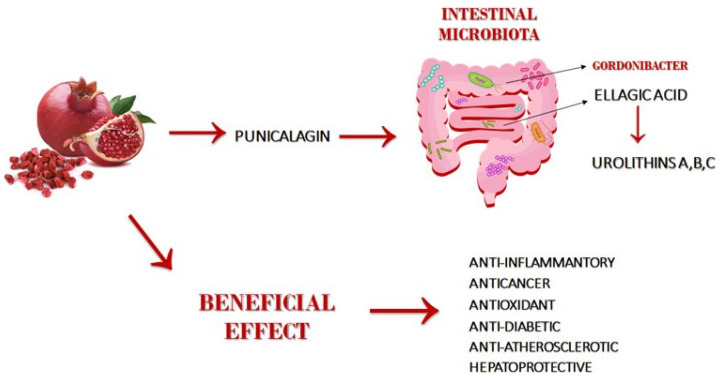
Metabolism of punicalagin and its beneficial effects.

PUN and its metabolites have been shown to inhibit angiogenesis, proliferation, and induce apoptosis in osteosarcoma cancer cells, prostate cancer, colon cancer and cervical cancer cell lines. PUN also suppresses various signaling pathways, including NF-kB, MAPK, Bcl-XL and LKB1-AMPK-p27, suggesting that PUN could have potential for therapeutics of various immune diseases, including cancer, atherosclerosis, hyperlipidemia, myocardial ischemia, diabetes, inflammation and infections, male infertility, brain damage, obesity and Alzheimer’s disease (Figure 1). For information summarizing in vitro, preclinical and interventional studies conducted with PUN, see Table 1.

The question of whether PUN, EA or urolithins are actually responsible for health benefits has not yet been clarified and is still under discussion. The fact is that virtually only urolithins are adsorbed, able to circulate in the blood and reach different target tissues, where they trigger different molecular and cellular reactions. They are believed to be a true bioactive compound. However, a solution to the bioavailability of PUN could be to reduce the molecule and encapsulate it. However, this requires further investigation into whether the biological effects of PUN, EA or urolithins reported in vitro may be of real significance in vivo.

## Figures and Tables

**Table 1:** Summary of the *in vitro*, preclinical and interventional studies involving punicalagin research.

In Vitro	Preclinical	Clinical/Interventional	Aim/Mechanism	References
KB and CAL27 oral cancer, SW480, SW620, HT29 and HCT116 colon cancer and RWPE-1 prostate cancer cell lines	-	-	Apoptotic and antioxidant activity	[8]
Vero (normal African green monkey kidney cell line), Hep-2 (human larynx epithelial cancer cell line), and A-549 (human small cell lung carcinoma cell line)	-	-	ROS elimination and antioxidant activity	[11]
HeLa cells	-	-	Antiproliferative activity (β-catenin signaling pathway)	[12]
Vero (African green monkey kidney cells, ATCC CCL-81), HEL (human embryonic lung fibroblast, ATCC CCL-137), and A549 (human lung carcinoma, ATCC CCL-185)	-	-	Antiviral activity	[14]
-	-	20 young healthy volunteers (10 men (BMI 21.8 ± 2.5 kg/m^2^) and 10 women (BMI 23.4 ± 1.6 kg/m), with an average age of 20.5 ± 2.0 and 21.5 ± 1.5 years	Bioavailability of PUN and EA	[20]
-	-	Healthy volunteers (*n* = 49, 32 men and 17 women; BMI > 27 kg/m^2^) aged between 40 and 65 years	Metabolism EA	[23]
-	Rat	-	Bioavailability and metabolism of PUN and EA	[26]
-	-	Healthy volunteers (*n* = 6, 4 men and 2 women)	Bioavailability and metabolism of PUN and EA	[27]
RAW264.7 cells	-	-	Anti-inflammatory activity	[32]
RAW264.7 cells	-	-	Anti-inflammatory activity	[33]
Splenocytes from Balb/c mice and normal splenocytes from C57 Bl/6 mice	Mouse Balb/c	-	Immunosuppressive activity	[34]
RAW264.7 cells	-	-	Anti-inflammatory activity	[35]
PBMCs from healthy volunteers	-	-	Anti-inflammatory activity	[36]
Jurkat E2 cells and PBMC	-	-	Immunosuppressive activity	[37]
Buňky (ME-180)—Cell carcinoma of the cervix uteri	-	-	Apoptotic activity through the mitochondrial and NF-kB pathway	[40]
-	Mouse (Swiss Webster)	-	Chemoprotective and angiogenic activity	[41]
human osteosarcoma cell lines (U2OS, MG63 and SaOS2) and normal osteoblast cell line (hFOB1.19)	-	-	Antiproliferative activity	[43]
human colon cell line Caco-2 and the normal colon cells CCD-112CoN	-	-	Apoptotic activity	[45]
human U87MG glioma cells	-	-	Apoptotic activity	[46]
Streptococcus mutans	-	-	Antibacterial activity	[49]
SARS-CoV-2 (spike protein)	-	-	Antiviral activity	[50]
-	Mouse Balb/c	-	Antioxidant activity	[54]
-	Male Rats (Sprague-Dawley)	-	Antihepatotoxicity	[57]
-	Male Wistar rats	-	Antihepatotoxicity and antineurotoxicity	[59]
3T3-L1 murine pre-adipocytes	-	-	Antiobesity activity	[60]
3T3-L1 mouse adipocytes	-	-	Antiobesity activity	[61]
Human adipocytes (SGBS), human myocytes (HSMM)	-	-	Antiobesity activity	[62]
Human Umbilical Vein Endothelial Cells (HUVECs)	-	-	Antiatherogenic effects	[63]
THP-1 cells (Human immortalized cells, ATCC, TIB-202)	Male apoE−/− mice	-	Anti-atherosclerotic activity	[65]
-	Wistar rats (*n* = 48)	-	Anti-atherosclerotic activity	[66]
-	Sprague−Dawley rats	-	Toxicity effect	[67]
Chinese hamster ovary (CHO) cells	Mouse Balb/c	-	Genotoxicity effect	[68]
Chinese hamster cells (B14 cell line)			Genotoxicity and cytotoxicity effect	[69]

ROS: oxygen-reactive substances, BMI: body mass index, PUN: Punicalagin, EA: ellagic acid, PBMCs: Peripheral blood mononuclear cells.

## References

[1] Jurenka J.S. (2008). Therapeutic applications of pomegranate (*Punica granatum* L.): A review. Altern. Med. Rev. A J. Clin..

[2] Abdollahzadeh S., Mashouf R., Mortazavi H., Moghaddam M., Roozbahani N., Vahedi M. (2011). Antibacterial and antifungal activ-ities of punica granatum peel extracts against oral pathogens. J. Dent..

[3] Syed D.N., Chamcheu J.-C., Adhami V.M., Mukhtar H. (2013). Pomegranate extracts and cancer prevention: Molecular and cellular activities. Anti-Cancer Agents Med. Chem..

[4] Paller C.J., Pantuck A., Carducci M.A. (2017). A review of pomegranate in prostate cancer. Prostate Cancer Prostatic Dis..

[5] Singh B., Singh J.P., Kaur A., Singh N. (2018). Phenolic compounds as beneficial phytochemicals in pomegranate (*Punica granatum* L.) peel: A review. Food Chem..

[6] Kraszni M., Marosi A., Larive C.K. (2013). NMR assignments and the acid–base characterization of the pomegranate ellagitannin punicalagin in the acidic pH-range. Anal. Bioanal. Chem..

[7] Gil M.I., Tomás-Barberán F.A., Hess-Pierce B., Holcroft D.M., Kader A.A. (2000). Antioxidant Activity of Pomegranate Juice and Its Relationship with Phenolic Composition and Processing. J. Agric. Food Chem..

[8] Seeram N.P., Adams L.S., Henning S.M., Niu Y., Zhang Y., Nair M.G., Heber D. (2005). *In vitro* antiproliferative, apoptotic and antioxi-dant activities of punicalagin, ellagic acid and a total pomegranate tannin extract are enhanced in combination with oth-er polyphenols as found in pomegranate juice. J. Nutr. Biochem..

[9] Oudane B., Boudemagh D., Bounekhel M., Sobhi W., Vidal M., Broussy S. (2018). Isolation, characterization, antioxidant activity, and protein-precipitating capacity of the hydrolyzable tannin punicalagin from pomegranate yellow peel (*Punica granatum*). J. Mol. Struct..

[10] Moilanen J., Karonen M., Tähtinen P., Jacquet R., Quideau S., Salminen J.-P. (2016). Biological activity of ellagitannins: Effects as an-ti-oxidants, pro-oxidants and metal chelators. Phytochemistry.

[11] Kulkarni A.P., Mahal H., Kapoor S., Aradhya S. (2007). *In vitro* studies on the binding, antioxidant, and cytotoxic actions of puni-calagin. J. Agric. Food Chem..

[12] Tang J., Li B., Hong S., Liu C., Min J., Hu M., Li Y., Liu Y., Hong L. (2017). Punicalagin suppresses the proliferation and invasion of cervical cancer cells through inhibition of the β-catenin pathway. Mol. Med. Rep..

[13] Bialonska D., Ramnani P., Kasimsetty S.G., Muntha K.R., Gibson G.R., Ferreira D. (2010). The influence of pomegranate by-product and punicalagins on selected groups of human intestinal microbiota. Int. J. Food Microbiol..

[14] Lin L.-T., Chen T.-Y., Lin S.-C., Chung C.-Y., Lin T.-C., Wang G.-H., Anderson R., Lin C.-C., Richardson C.D. (2013). Broad-spectrum antiviral activity of chebulagic acid and punicalagin against viruses that use glycosaminoglycans for entry. BMC Microbiol..

[15] Zahin M., Ahmad I., Gupta R.C., Aqil F. (2014). Punicalagin and Ellagic Acid Demonstrate Antimutagenic Activity and Inhibition of Benzo[a]pyrene Induced DNA Adducts. Biomed. Res. Int..

[16] Heber D., Benzie I.F.F., Wachtel-Galor S. (2011). Pomegranate Ellagitannins. Herbal Medicine: Biomolecular and Clinical Aspects.

[17] Espín J.C., Larrosa M., García-Conesa M.T., Tomás-Barberán F. (2013). Biological Significance of Urolithins, the Gut Microbial Ellagic Acid-Derived Metabolites: The Evidence So Far. Evid. Based Complementary Altern. Med..

[18] Silacci P., Tretola M. (2019). Pomegranate’s Ellagitannins: Metabolism and Mechanisms of Health Promoting Properties. Nutr. Food Sci. Int. J..

[19] García-Villalba R., Vissenaekens H., Pitart J., Vaquero M.R., Espín J.C., Grootaert C., Selma M.V., Raes K., Smagghe G., Possemiers S. (2017). Gastrointestinal Simulation Model TWIN-SHIME Shows Differences between Human Urolithin-Metabotypes in Gut Microbiota Composition, Pomegranate Polyphenol Metabolism, and Transport along the Intestinal Tract. J. Agric. Food Chem..

[20] González-Sarrías A., García-Villalba R., Núñez-Sánchez Á.M., Tomé-Carneiro J., Zafrilla P., Mulero J., Tomás-Barberán F.A., Espín J.C. (2015). Identifying the limits for ellagic acid bioavailability: A crossover pharmacokinetic study in healthy volunteers after consumption of pomegranate extracts. J. Funct. Foods.

[21] Selma M.V., Tomas-Barberan F.A., Beltran D., García-Villalba R., Espín J.C. (2014). Gordonibacter urolithinfaciens sp. nov., a uro-lithin-producing bacterium isolated from the human gut. Int. J. Syst. Evol. Microbiol..

[22] Selma M.V., Beltrán D., García-Villalba R., Espín J.C., Tomás-Barberán F.A. (2014). Description of urolithin production capacity from ellagic acid of two human intestinal *Gordonibacter* species. Food Funct..

[23] Vaquero M.R., García-Villalba R., González-Sarrías A., Beltrán D., Tomás-Barberán F.A., Espín J.C., Selma M.V. (2015). Interindividual variability in the human metabolism of ellagic acid: Contribution of *Gordonibacter* to urolithin production. J. Funct. Foods.

[24] Selma M.V., Romo-Vaquero M., García-Villalba R., González-Sarrías A., Tomás-Barberán F.A., Espín J.C. (2015). The human gut microbial ecology associated with overweight and obesity determines ellagic acid metabolism. Food Funct..

[25] Qin G., Xu C., Ming R., Tang H., Guyot R., Kramer E.M., Hu Y., Yi X., Qi Y., Xu X. (2017). The pomegranate (*Punica granatum* L.) genome and the genomics of punicalagin biosynthesis. Plant J..

[26] Llorach R., Cerdá B., Cerón J.J., Espín J.C., Tomás-Barberán F.A. (2003). Evaluation of the bioavailability and metabolism in the rat of punicalagin, an antioxidant polyphenol from pomegranate juice. Eur. J. Nutr..

[27] Cerdá B., Espín J.C., Parra S., Martínez P., Tomás-Barberán F.A. (2004). The potent *in vitro* antioxidant ellagitannins from pomegranate juice are metabolised into bioavailable but poor antioxidant hydroxy–6H–dibenzopyran–6–one derivatives by the colon-ic microflora of healthy humans. Eur. J. Nutr..

[28] Zuccari G., Baldassari S., Ailuno G., Turrini F., Alfei S., Caviglioli G. (2020). Formulation Strategies to Improve Oral Bioavailability of Ellagic Acid. Appl. Sci..

[29] Nyamba I., Lechanteur A., Semdé R., Evrard B. (2020). Physical formulation approaches for improving aqueous solubility and bio-availability of ellagic acid: A review. Eur. J. Pharm. Biopharm..

[30] Williams H.D., Trevaskis N.L., Charman S., Shanker R.M., Charman W., Pouton C., Porter C.J.H. (2013). Strategies to Address Low Drug Solubility in Discovery and Development. Pharm. Rev..

[31] Qu W., Iii A.P.B., Pan Z., Ma H. (2012). Quantitative determination of major polyphenol constituents in pomegranate products. Food Chem..

[32] Nathan C. (2002). Points of control in inflammation. Nat. Cell Biol..

[33] Xu X., Yin P., Wan C., Chong X., Liu M., Cheng P., Chen J., Liu F., Xu J. (2014). Punicalagin inhibits inflammation in LPS-induced RAW264.7 macrophages via the suppression of TLR4-mediated MAPKs and NF-κB activation. Inflammation.

[34] BenSaad L.A., Kim K.H., Quah C.C., Kim W.R., Shahimi M. (2017). Anti-inflammatory potential of ellagic acid, gallic acid and puni-calagin A&B isolated from Punica granatum. BMC Complementary Altern. Med..

[35] Lee S.-I., Kim B.-S., Kim K.-S., Lee S., Shin K.-S., Lim J.-S. (2008). Immune-suppressive activity of punicalagin via inhibition of NFAT activation. Biochem. Biophys. Res. Commun..

[36] Cao Y., Chen J., Ren G., Zhang Y., Tan X., Yang L. (2019). Punicalagin Prevents Inflammation in LPS-Induced RAW264.7 Macro-phages by Inhibiting FoxO3a/Autophagy Signaling Pathway. Nutrients.

[37] Ngkelo A., Meja K., Yeadon M., Adcock I., Kirkham P.A. (2012). LPS induced inflammatory responses in human peripheral blood mononuclear cells is mediated through NOX4 and Giα dependent PI-3kinase signalling. J. Inflamm..

[38] Trevillyan J.M., Chiou X.G., Chen Y.-W., Ballaron S.J., Sheets M.P., Smith M.L., Wiedeman P.E., Warrior U., Wilkins J., Gubbins E.J. (2001). Potent Inhibition of NFAT Activation and T Cell Cytokine Production by Novel Low Molecular Weight Pyrazole Compounds. J. Biol. Chem..

[39] Rahimi H.R., Arastoo M., Ostad S.N. (2012). A Comprehensive Review of *Punica granatum* (Pomegranate) Properties in Toxicolog-ical, Pharmacological, Cellular and Molecular Biology Researches. Iran J. Pharm. Res..

[40] Elmore S. (2007). Apoptosis: A review of programmed cell death. Toxicol. Pathol..

[41] Zhang L., Chinnathambi A., Alharbi S.A., Veeraraghavan V.P., Mohan S.K., Zhang G. (2020). Punicalagin promotes the apoptosis in human cervical cancer (ME-180) cells through mitochondrial pathway and by inhibiting the NF-kB signaling pathway. Saudi J. Biol. Sci..

[42] Carneiro C.C., Santos S., Lino R.D.S., Bara M.T.F., Chaibub B.A., Reis P.R.D.M., Chaves D.A., da Silva A.J.R., Silva L.S., Silva D.D.M.E. (2016). Chemopreventive effect and angiogenic activity of punicalagin isolated from leaves of *Lafoensia pacari* A. St.-Hil. Toxicol. Appl. Pharm..

[43] Stahlhut C., Slack F.J. (2013). MicroRNAs and the cancer phenotype: Profiling, signatures and clinical implications. Genome Med..

[44] Huang T., Zhang X., Wang H. (2020). Punicalagin inhibited proliferation, invasion and angiogenesis of osteosarcoma through suppression of NF-κB signaling. Mol. Med. Rep..

[45] Syed D.N., Malik A., Hadi N., Sarfaraz S., Afaq F., Mukhtar H. (2006). Photochemopreventive Effect of Pomegranate Fruit Extract on UVA-mediated Activation of Cellular Pathways in Normal Human Epidermal Keratinocytes. Photochem. Photobiol..

[46] Larrosa M., Tomás-Barberán F.A., Espín J.C. (2006). The dietary hydrolysable tannin punicalagin releases ellagic acid that induces apoptosis in human colon adenocarcinoma Caco-2 cells by using the mitochondrial pathway. J. Nutr. Biochem..

[47] Wang S.G., Huang M.H., Li J.H., Lai F.I., Lee H.M., Hsu Y.N. (2013). Punicalagin induces apoptotic and autophagic cell death in human U87MG glioma cells. Acta Pharm. Sin..

[48] Liang J., Shao S.H., Xu Z.-X., Hennessy B., Ding Z., Larrea M., Kondo S., Dumont D.J., Gutterman J.U., Walker C.L. (2007). The energy sensing LKB1–AMPK pathway regulates p27kip1 phosphorylation mediating the decision to enter autophagy or apoptosis. Nat. Cell Biol..

[49] Ammar O.M.A., Ilktac M., Gülcan H. (2019). Urolithins and their antimicrobial activity: A short review. EMU J. Pharm. Sci..

[50] Gulube Z., Patel M. (2016). Effect of Punica granatum on the virulence factors of cariogenic bacteria Streptococcus mutans. Microb. Pathog..

[51] Tito A., Colantuono A., Pirone L., Pedone E., Intartaglia D., Giamundo G., Conte I., Vitaglione P., Apone F. (2021). A pomegranate peel extract as inhibitor of SARS-CoV-2 Spike binding to human ACE2 (*in vitro*): A promising source of novel antiviral drugs. Front. Chem..

[52] Pizzino G., Irrera N., Cucinotta M., Pallio G., Mannino F., Arcoraci V., Squadrito F., Altavilla D., Bitto A. (2017). Oxidative Stress: Harms and Benefits for Human Health. Oxidative Med. Cell. Longev..

[53] Djedjibegovic J., Marjanovic A., Panieri E., Saso L. (2020). Ellagic Acid-Derived Urolithins as Modulators of Oxidative Stress. Oxidative Med. Cell. Longev..

[54] Liguori I., Russo G., Curcio F., Bulli G., Aran L., DELLA Morte D., Gargiulo G., Testa G., Cacciatore F., Bonaduce D. (2018). Oxidative stress, aging, and diseases. Clin. Interv. Aging.

[55] Sun Y.Q., Xin T.A.O., Men X.M., Xu Z.W., Tian W.A.N.G. (2017). *In vitro* and in vivo antioxidant activities of three major polyphenolic com-pounds in pomegranate peel: Ellagic acid, punicalin, and punicalagin. J. Integr. Agric..

[56] Wang Y., Zhang H., Liang H., Yuan Q. (2013). Purification, antioxidant activity and protein-precipitating capacity of punicalin from pomegranate husk. Food Chem..

[57] Bialonska D., Kasimsetty S.G., Khan S.I., Ferreira D. (2009). Urolithins, Intestinal Microbial Metabolites of Pomegranate Ellagitannins, Exhibit Potent Antioxidant Activity in a Cell-Based Assay. J. Agric. Food Chem..

[58] Fouad A.A., Qutub H.O., Al-Melhim W.N. (2016). Punicalagin alleviates hepatotoxicity in rats challenged with cyclophosphamide. Environ. Toxicol. Pharmacol..

[59] Luedde T., Schwabe R.F. (2011). NF-κB in the liver—linking injury, fibrosis and hepatocellular carcinoma. Nat. Rev. Gastro-Enterol. Hepatol..

[60] Foroutanfar A., Mehri S., Marzyeh K., Tandisehpanah Z., Hosseinzadeh H. (2020). Protective effect of punicalagin, the main poly-phenol compound of pomegranate, against acrylamide-induced neurotoxicity and hepatotoxicity in rats. Phytother. Res..

[61] Les F., Arbonés-Mainar J.M., Valero M.S., López V. (2018). Pomegranate polyphenols and urolithin A inhibit α-glucosidase, dipeptidyl peptidase-4, lipase, triglyceride accumulation and adipogenesis related genes in 3T3-L1 adipocyte-like cells. J. Ethnopharmacol..

[62] Wu D., Ma X., Tian W. (2013). Pomegranate husk extract, punicalagin and ellagic acid inhibit fatty acid synthase and adipogenesis of 3T3-L1 adipocyte. J. Funct. Foods.

[63] Reguero M., Gómez de Cedrón M., Reglero G., Quintela J.C., de Molina A.R. (2021). Natural Extracts to Augment Energy Ex-penditure as a Complementary Approach to Tackle Obesity and Associated Metabolic Alterations. Biomolecules.

[64] Mele L., Mena P., Piemontese A., Marino V., López-Gutiérrez N., Bernini F., Brighenti F., Zanotti I., Del Rio D. (2016). Antiatherogenic effects of ellagic acid and urolithins *in vitro*. Arch. Biochem. Biophys..

[65] Kruth S.H. (2013). Fluid-phase pinocytosis of LDL by macrophages: A novel target to reduce macrophage cholesterol accumula-tion in atherosclerotic lesions. Curr. Pharm. Des..

[66] Zhao W., Wang L., Haller V., Ritsch A. (2019). A Novel Candidate for Prevention and Treatment of Atherosclerosis: Urolithin B Decreases Lipid Plaque Deposition in apoE(-/-) Mice and Increases Early Stages of Reverse Cholesterol Transport in ox-LDL Treated Macrophages Cells. Mol. Nutr. Food Res..

[67] Cui G.-H., Chen W.-Q., Shen Z.-Y. (2018). Urolithin A shows anti-atherosclerotic activity via activation of class B scavenger receptor and activation of Nef2 signaling pathway. Pharm. Rep..

[68] Cerdá B., Cerón J.J., Tomás-Barberán F.A., Espín J.C. (2003). Repeated oral administration of high doses of the pomegranate ellag-itannin punicalagin to rats for 37 days is not toxic. J. Agric. Food Chem.

[69] Sánchez-Lamar A., Fonseca G., Fuentes J.L., Cozzi R., Cundari E., Fiore M., Ricordy R., Perticone P., Degrassi F., De Salvia R. (2008). As-sessment of the genotoxic risk of *Punica granatum* L. (Punicaceae) whole fruit extracts. J. Ethnopharmacol..

[70] Labieniec M., Gabryelak T. (2003). Effects of tannins on Chinese hamster cell line B14. Mutat. Res. Toxicol. Env. Mutagen..

